# Editorial: Biomolecular solid-state NMR: Methods and applications

**DOI:** 10.3389/fmolb.2022.1082067

**Published:** 2022-12-08

**Authors:** Amir Goldbourt, Loren B. Andreas, Józef R. Lewandowski

**Affiliations:** ^1^ School of Chemistry, Tel Aviv University, Tel Aviv, Israel; ^2^ Department for NMR-Based Structural Biology, Max Planck Institute for Multidisciplinary Sciences, Göttingen, Germany; ^3^ Department of Chemistry, University of Warwick, Coventry, United Kingdom

**Keywords:** magic angle spinning (MAS), nuclear magnetic resonance (NMR), spectroscopy, protein assemblies, protein dynamics, carbohydrates, dipolar recoupling methods, amyloid fibrils, RNA, membrane proteins

The field of biomolecular solid-state nuclear magnetic resonance (ssNMR) has matured in the last two decades allowing the structural and dynamical characterization of highly complex systems down to atomic resolution. This special issue shows a collection of original studies as well as reviews from leading experts in the field that both advance the methodology, and also span many of the topics where ssNMR makes an impact in structural and functional biology.

A real strength of ssNMR is an ability to structurally characterize not only samples with long range molecular order such as crystals but also systems with short range order like fibrils and membrane proteins or even disordered systems. The applicability of ssNMR is continuously extended by active method development. One of the breakthroughs in ssNMR was the advent of fast sample spinning, which is constantly redefined with maximum frequencies exceeding 60 kHz, then 100 kHz and, recently, even 170 kHz. In this context, Duong et al. showed how spinning between 60–70 kHz allows the measurement of selective ^1^H-^14^N distances by exciting and detecting protons that are coupled to ^14^N, which provides very useful information for biological systems without isotopic labelling. In the study dipolar recoupling is achieved by saturating the overtone transitions of the natural abundant nitrogen spins. In the example of Paluch et al. several ^13^C homonuclear mixing schemes are systematically compared with the aim to facilitate assignment of methyl groups in high-molecular weight proteins. The authors demonstrated dramatic improvements in methyl to alpha carbon transfer efficiencies upon increasing from 55 to 95 kHz spinning rates. In another contribution to this issue, Zadorozhnyi et al. described a spectral editing technique to determine histidine protonation states, which play important functional roles in proton transfer, metal binding *etc.* The authors demonstrated how highly resolved information can be obtained using ^1^H detection within 2D experiments based on selective inversion of ring nitrogen atoms. To contrast the methodology relying on fast spinning, van der Wel comprehensively reviews moderate spinning rates based methods for measuring dihedral angles in peptides and proteins as a complement to more commonly recorded distance restraints. The emphasis is on direct measurements *via* correlations of anisotropic interactions including ϕ and ψ backbone dihedral angles *via* HNCH and NCCN experiments, sidechain angles *via* HCCH, the peptide bond angle ω, and long-range angle restraints between backbone amides. The review contains various examples on real systems, including amyloids, and details on the particularity of the pulse programs.

Several interesting applications can be viewed in this special issue. A field to which ssNMR has been continuously contributing key structural and functional information is that of amyloids and other protein aggregation phenomena. Qi et al. provide an excellent example for the complementarity and contribution of ssNMR studies to our understanding of fibril formation. Since ssNMR does not require long-range crystallinity of the samples, they have been able to study variants of the Y145Stop mutant of the human prion protein, which is associated with hereditary prionopathy. The variants were made of different deletions in the flexible N-terminal tail, and the authors have been able to show how those deletions affect or maintain the aggregation properties of the protein on an atomic level. In the review of Kitoka et al., many aspects of the tau protein, as viewed by NMR and in particular ssNMR spectroscopy, are discussed. The tau protein forms intracellular neurofibrillary tangles in neurons, and is a major drug target to treat Alzheimer’s disease. The review describes solution NMR efforts to study the monomeric form, its secondary structure, and the effects of phosphorylation on aggregation properties. Solid-state NMR studies, including dynamic nuclear polarization (DNP), contributed to our understanding of oligomer and filamentous structures of various tau constructs including a three-dimensional structural model of its fold in the fibrillar form.

An additional application area to which ssNMR techniques have significantly contributed is the study of membrane proteins in close to native environments, and in particular it is possible to study them in membrane bilayers. Amani et al. contributed a structural study of the potassium channel KirBac1.1 from the bacterium *Burkholderia pseudomallei* that causes Melioidosis. An original X-ray structure lacked 85 residues of the total of the 333, mainly in the N- and C-terminus regions. Using T2-filtered ssNMR experiments, the authors generated surface accessibility potentials based on the assumption that only those residues in the vicinity of water can be detected.

Yet another field where ssNMR has increasing impact is study of biomolecular complexes. Recent progress in the study of RNA and ribonucleic acid–protein complexes (RNPs) are discussed in detail by Aguion and Marchanka. This unique review discusses strategies to label synthetic RNA, means to assign RNA polynucleotides (including ^1^H spins), and dedicates attention to discussions on the complexities and possible opportunities. Discussions on the particular stages in assigning ribose, base, their linkage, and sequential contacts are highly detailed and accompanied by many examples, pulse sequence details, and spectra. One of the key protein-DNA complexes in the cell is chromatin, consisting of the DNA wrapped with histone proteins. Ackermann and Debelouchina describe the emerging contributions of ssNMR to understanding this complex and highly important gene expression system. They discuss the details of histone preparation and isotopic labeling strategies including DNA and the four histones, as well as post translational modifications, techniques and results from studies of the rigid core and flexible histone tails, and chromatin modulators. While chromatin studies by NMR are highly complex, the set of biochemical and NMR tools presented in this review will help to advance further understanding of chromatin structural biology.

Given that a majority of the applications in the field of biomolecular ssNMR focus on proteins, and recently more studies on polynucleotides emerge (see review below), it is interesting to see that significant progress is also achieved to study the complex polysaccharide networks making up cell walls. Fernando et al. studied the polymorphism of carbohydrates making up the fungal cell wall. They find that the chitin moiety shows similarity to the α- and γ-allomorphs and is not significantly altered in the presence of anti-fungal treatment. In addition, statistical analysis revealed that chitosan (a deacetylation product of chitin) from *R. delemar* and *A. sydowii* share some similarity to Type-II chitosan (a relaxed two-fold helix conformation) but is completely different from Type-I.

Besides being a tool for structural characterization ssNMR is also important for its ability to probe dynamics spanning several orders of magnitude in time scale on challenging systems including fibrils, membrane proteins and large biomolecular complexes. To obtain a comprehensive view of the molecular motions in different regimes requires different complementary methods. For example, Vugmeyster et al. demonstrated how slow motions (in the order or 10^4^–10^5^ s^−1^) can be probed by utilizing ^2^H Chemical Exchange Saturation Transfer (CEST) techniques, both at slow and fast MAS rates. In another contribution, Franks et al. show how at fast spinning NH dipolar couplings can be measured using newly optimized symmetry-based pulses, previously utilized mostly at moderate spinning of 10–30 kHz, to enable such measurements for large protein complexes requiring high sensitivity afforded by proton detected experiments at fast spinning. Dipolar couplings report on cumulative amplitudes of motion for picosecond to microsecond motions and thus valuable parameters for characterizing dynamics on their own but also often employed to restrain overall motional amplitudes in model-free types of analyses of relaxation rates. In a related context, Zumpfe and Smith provide an insightful review of methods to quantify protein dynamics based on relaxation rate measurements in the solid state. They consider the model-free, extended model-free, spectral density mapping, and the LeMaster’s approaches highlighting their advantages, disadvantages and pitfalls that can lead to erroneous interpretation of molecular motions. The authors then show the advantage of the detectors method, in particular its generality and its use to describe molecular dynamics (MD) and thus extend our correlation of NMR and MD simulation data.

Two additional contributions demonstrate the strength of combining ssNMR with dynamic nuclear polarization (DNP) to study in-cell NMR. While this field is still in its infancy, studies slowly reveal both the technicalities and the advantages of such experiments that have to be performed at low temperatures (∼100 K) and with radicals. For example, Overall and Barnes discuss the effects of DNP radicals and cryoprotectants on cell viability (using human Jurkat cells) and signal enhancement showing that 10% d_6_-DMSO maintains the same enhancement as “DNP juice” (60/30/10 d_8_-glycerol/D_2_O/H_2_O) motivated by the superiority of DMSO with respect to the conditions of the cells. Very similarly, Xiao et al. has shown that in Human embryonic kidney 293 (HEK293) cells, incubation with the radical AMUPol and using 10% DMSO as a cryoprotectant along with slow cooling, were essential for cell integrity and provided similar enhancements as 15% glycerol. It was also shown that distribution of the radicals within the cells was non-uniform.

Overall, this special issue covers a large variety of topics providing insight into the diversity of applications of ssNMR, the state-of-the-art technology, and the wide range of experimental approaches that are available and that continuously extend to fit new applications and new needs.

